# RAGE and its ligands in breast cancer progression and metastasis

**DOI:** 10.3389/or.2024.1507942

**Published:** 2025-01-03

**Authors:** Madalina Coser, Bogdan Mihai Neamtu, Bogdan Pop, Calin Remus Cipaian, Maria Crisan

**Affiliations:** ^1^ Department of Histology, Doctoral School “Iuliu Hatieganu” University of Medicine and Pharmacy Cluj-Napoca, Cluj-Napoca, Romania; ^2^ Clinical Medical Department, Center for Research in Mathematics and Applications, Faculty of Medicine, “Lucian Blaga” University Sibiu, Sibiu, Romania; ^3^ Department of Clinical Research, Pediatric Clinical Hospital Sibiu, Sibiu, Romania; ^4^ Department of Pathology, “Iuliu Hatieganu” University of Medicine and Pharmacy Cluj-Napoca, Cluj-Napoca, Romania; ^5^ Department of Pathology, “Prof. Dr. ion Chiricuta” Institute of Oncology Cluj-Napoca, Cluj-Napoca, Romania; ^6^ Second Medical Clinic, Sibiu County Clinical Emergency Hospital, Sibiu, Romania; ^7^ Clinical Medical Department, Faculty of Medicine, “Lucian Blaga” University Sibiu, Sibiu, Romania; ^8^ Department of Histology, “Iuliu Hatieganu” University of Medicine and Pharmacy Cluj-Napoca, Cluj-Napoca, Romania; ^9^ Clinic of Dermatology, Emergency Clinical County Hospital, Cluj-Napoca, Romania

**Keywords:** RAGE, AGEs, breast cancer, S100, HMGB1, metastasis, lifestyle

## Abstract

**Introduction:**

Breast cancer is the most common form of cancer diagnosed worldwide and the leading cause of death in women globally, according to Globocan 2020. Hence, investigating novel pathways implicated in cancer progression and metastasis could lead to the development of targeted therapies and new treatment strategies in breast cancer. Recent studies reported an interplay between the receptor for advanced glycation end products (RAGE) and its ligands, S100 protein group, advanced glycation end products (AGEs) and high-mobility group box 1 protein (HMGB1) and breast cancer growth and metastasis.

**Materials and methods:**

We used articles available in the NCBI website database PubMed to write this scoping review. The search words used were ‘RAGE receptor’ AND/OR ‘breast cancer, RAGE ligands, glycation end products’. A total of 90 articles were included. We conducted a meta-analysis to assess the relationship between the RAGE rs1800624 polymorphism and breast cancer risk using fixed-effect or random-effect models to calculate odds ratios (ORs) and their corresponding 95% confidence intervals (95% CIs).

**Results:**

RAGE upon activation by its ligands enhances downstream signaling pathways, contributing to breast cancer cells migration, growth, angiogenesis, metastasis, and drug resistance. In addition, studies have shown that RAGE and its ligands influence the way breast cancer cells interact with immune cells present in the tumor microenvironment (macrophages, fibroblasts), thus regulating it to promote tumor growth and metastasis.

**Conclusion:**

Breast cancers with a high expression of RAGE are associated with poor prognosis. Targeting RAGE and its ligands impairs cell invasion and metastasis, showing promising potential for further research as potential prognostic biomarkers or targeted onco-therapeutics.

## 1 Introduction

This scoping review addresses both old and recent information regarding the implications of the receptor for advanced glycation end-products (RAGE) and its multiple ligands in breast cancer progression and metastasis and their potential to become future biomarkers or therapeutic targets. We will summarize the valuable preclinical and clinical evidence available on RAGE expression in breast cancer cells, interactions with its major ligands and the activation of downstream signaling pathways and their potential as therapeutic targets.

Breast cancer is a major public health concern worldwide with approximately 2.26 million new cases and 680 thousand new deaths in 2020 (1). Moreover, it is a heterogeneous malignancy classified into distinct subtypes according to immunohistochemistry markers: estrogen receptor (ER), progesterone receptor (PR) and human epidermal growth factor receptor-2 (HER2). Triple negative (basal like) subtype, which lacks the expression of all the immunochemistry markers, accounts for 10%–17% of all breast cancers and has a poor prognosis due to high proliferation and aggressive clinical behavior. 70% are ER positive (luminal subtypes), showing a better prognosis than the other subtypes. HER2 overexpressing subtypes account for 18%–25% of breast cancers presenting poor differentiation and worse prognosis than luminal subtypes (2–5). Metastasis remains a major cause of mortality in breast cancer patients. Therefore, it is important to identify mechanisms that regulate progression, invasion and metastasis along with the development of new targeted therapies (3).

Generally, cancer cells are inclined towards an aerobic metabolism of glucose, known as Warburg effect (6, 7). To meet the energy requirements associated with increased proliferation and compensate for an inefficient energy supply, cancer cells create a hyperglycemic microenvironment prone to increased glycation, oxidative stress and inflammation (4, 7). Advanced glycation end products (AGEs) are by-products of enhanced glycolytic flux implicated in the progression and invasion of cancers (4, 7). The biological effects of AGEs are mainly mediated by the binding to RAGE, which is a multiligand single transmembrane receptor and a member of the immunoglobulin superfamily of surface molecules expressed in both normal and cancer cells (4, 7–9). Protein glycation interferes with normal protein function therefore, AGEs exert direct damage to protein structures and extracellular matrix modification (7). In addition to AGEs, RAGE binds to a broad repertoire of ligands, which include: β2 integrin/Mac-1, amyloid β-peptide, damage-associated pattern molecules (DAMPs) - high-mobility group box protein 1 (HMGB1)/amphoterin and S100 group of proteins/calgranulines (10, 11). Upon binding to ligands, RAGE activates downstream signaling pathways, such as PI3K/AKT (phosphatidyl inositol-3-kinase/protein kinase B), JAK/STAT (Janus kinase/signal transducers and activators of transcription), RAS/MAPK (small GTPase binding protein/mitogen-activated protein kinase) and transcription factors, such as nuclear factor-kappa B (NF-kB), signal transducers and activators of transcription 3 (STAT3) and hypoxia inducible factor-1 α (HIF-1α), that augment and maintain chronic inflammatory conditions, which in turn enhance the progression of various cancers, including breast cancer (7–11).

It is now accepted that most solid tumors, including those in the breast, have an inflammatory microenvironment. A growing body of evidence shows that RAGE is an important mediator between the cancer cells and the components of the surrounding microenvironment, thus connecting chronic inflammation to neoplastic progression through various autocrine and/or paracrine interactions (3, 9).

To provide a more thorough understanding of the role of RAGE in the inflammatory microenvironment of breast tumors, we conducted an extensive literature review as outlined in the methods section.

## 2 Methods

**FIGURE 1:**
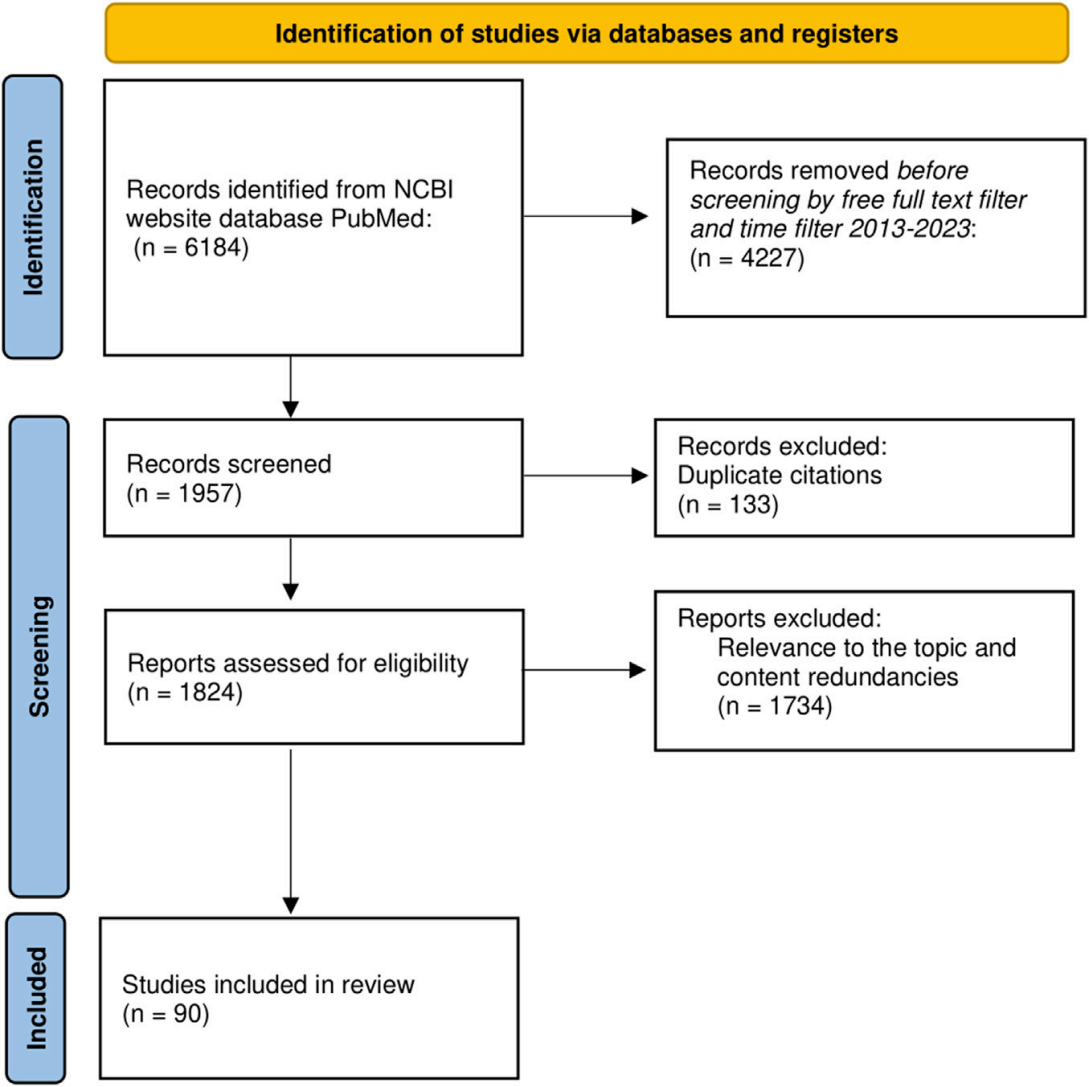
PRISMA flow diagram of literature search and article selection process. N = number of articles.

To elaborate this scoping literature review (12), we used open access articles available in the NCBI website database PubMed “https://www.ncbi.nlm.nih.gov/PubMed/”. By using MeSH terms and the advanced search builder of the database, we selected the main keyword “RAGE receptor”, followed by AND/OR “breast cancer, AGEs, advanced glycation end products, HMGB1, S100 proteins, RAGE ligands”. We incorporated articles published in the last 10 years, using all fields of search and focusing mainly on more recent articles published in 2019–2023. The main search using various combinations of the keywords without additional filters revealed 6,184 articles. By using Publishing Dates from 2013 to 2023 and the free full text filter, the search revealed 1,957 publications. After removing duplicate citation, 1,824 were assessed for eligibility. Other inclusion criteria were total citing of the articles, the impact factor of the published research, English language, peer reviewed, recent information and relevance to the topic. We excluded articles with unclear methodology, content redundancies and non-open access. The incorporated articles were experimental research, systematic reviews, narrative reviews and meta-analysis. Experimental studies used murine models, human breast cancer cell lines cultures, human fresh frozen tissue or paraffin imbedded tissue. The total number of articles included was 90, as shown in Figure 1.

The relationship between BC risk and RAGE gene polymorphism (*rs 1800624*), following genetic model of homozygous (AA vs. TT), heterozygous (AT vs. TT), dominant (AA + AT vs. TT) and recessive (AA vs. AT + TT) (13), was evaluated using ORs and 95% CIs. A *Z* test was used to assess the significance of overall ORs with a *P*-value of <0.5 considered statistically significant, alongside chi-square Q test and *I* ^*2*^ statistic for assessing heterogeneity as in previous studies (13). A *P*-value < 0.5 or *I* ^*2*^ > 50% indicated significant heterogeneity. In the presence of heterogeneity outcomes were determined using random effects, in addition to fixed effects model. The Restricted Maximum Likelihood (REML) estimation method was employed to account for heterogeneity in the meta-analysis outcomes. Additionally, the fixed-effects model, calculated using the Mantel-Haenszel method, was applied for pooled effect estimates. Egger’s test was conducted to assess potential publication bias in the included studies, with a significance level set at p < 0.05. All the tests were performed using SPSS software version 29 (Statistical Package for the Social Sciences). Our analysis required no ethical approval and patient consent, being based on previously published data.

With the analysis completed, we proceed to outline the results obtained from our study.

## 3 Results

### 3.1 Receptor for advanced glycation end products (RAGE)

RAGE is a single transmembrane, multiligand receptor of the immunoglobulin superfamily, encoded on the short arm of chromosome 6 (6p21.3), in the) class III region of the major histocompatibility complex (MHC), concerned with immune response (10, 11). It is expressed on a wide variety of cells, such as immune cells (macrophages), neurons, activated endothelial and vascular smooth muscle cells and cancer cells (including breast cancer) (2).

Innate immunity plays a crucial role in the response to dying and modified cells (e.g., cancer cells) (10, 14, 15). Apoptotic cells induce an immunosuppressive signal, avoiding the initiation of an autoimmune response, whereas necrotic cells activate innate immunity and induce inflammation by releasing DAMPS (10, 15). These endogenous danger molecules are then recognized by pattern recognition receptors (PRRs) (11, 14). RAGE is a PRR capable of recognizing these molecules associated with tissue damage (DAMP) (10). Although, initially reported as the receptor for AGEs (16), RAGE may be bound by many ligands which include extracellular HMGB 1 (the prototypic DAMP), members of the S100 family, β2 integrin/Mac-1, amyloid β-peptide and glycosaminoglycans (2, 10, 11). These molecules are abundant in the tumor microenvironment of most solid tumors (16).

**FIGURE 2:**
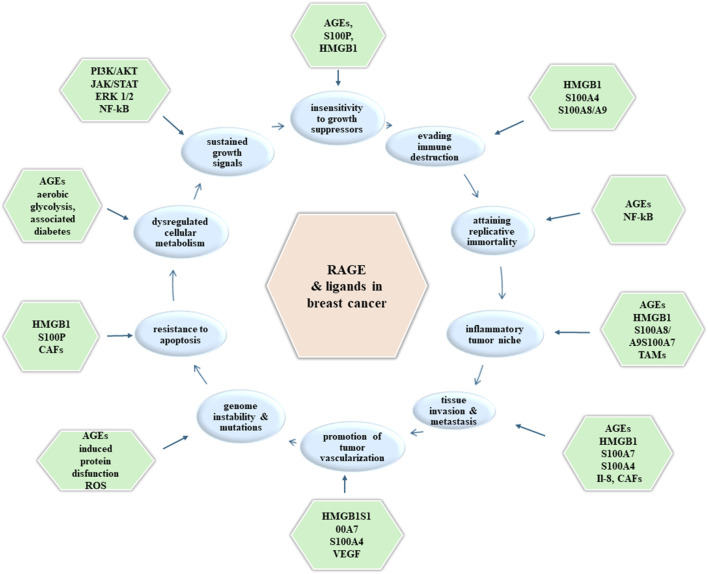
Molecules (outer boxes) involved in promoting each hallmark of cancer (inner boxes) upon RAGE activation in breast cancer tumorigenesis.

Activation of RAGE by its ligands initiates complex signaling pathways. The activation of NADPH oxidase, PI3K/AKT, JAK/STAT, NF-kB, Ras/MAPK, Rac1/cdc42p44/p42, p38 and SAP/JNK MAPK generates crucial down streaming inflammatory consequences. These include the activation of transcription factors NF-kB, activator protein (AP)-1 and STAT-3 (6, 11, 16). RAGE may also play an important role in cell adhesion and clustering as well as recruitment of inflammatory cells, while serving as a counter-receptor for leukocyte integrins (β-2 integrins) (10) (shown in Figure 2).

#### 3.1.1 RAGE gene polymorphism in breast cancer

There are several studies which investigated the relationship between RAGE gene polymorphism and breast cancer risk. RAGE genomic sequence is highly polymorphic and the ultimate genetic underpinnings of breast cancer remain a challengeable task (17).

In 2014 Pan et al (17) evaluated the association of four common polymorphisms (*rs1800625, rs1800624, rs2070600 and rs184003*) in RAGE gene and the risk of breast cancer in northeastern Han Chinese. The relatively small sample size study (509 breast cancer patients, 504 cancer-free controls) revealed that the *rs184003* TT genotype or T alle was overexpresses in patients relative to controls with a 1.62-fold increase in breast cancer risk for individuals carrying this alle. Another study suggested that polymorphic variants of RAGE *-374T/A (rs1800624)* decreased the risk of breast cancer among Chinese women (18). These findings were also reported in a meta-analysis, focused on the Asian population (19). However, by studying three polymorphisms of RAGE (*rs1800624, rs1800625, rs2070600*), Yue et al observed in the Han Chinese population a significant association between RAGE gene *rs1800624* polymorphism and breast cancer risk, with a cumulative impact of multiple risk associated with polymorphisms in this pathway on the development of breast cancer (20). This association was not reported in Pan et al ‘s previous study with a similar cohort. In the study by Tesarova et al (21), *rs2070600* RAGE polymorphism demonstrated negative prognostic value in terms of mortality due to breast cancer, in a cohort of Caucasian patients.

A more recent meta-analysis concluded that the RAGE *rs1800624* polymorphism may increase the risk of breast cancer in homozygous genetic model, especially in the Asian population, whereas the dominant model of *rs1800624* polymorphism may decrease breast cancer risk in the Caucasian population (13). Although, Peng et al did not observe a significant contribution of genetic variants to breast cancer risk, the population carrying haplotypes TA and TT (polymorphisms *rs2070600* and *rs1800624*) demonstrated increased breast cancer risk with higher levels of AGEs at the combined analysis of haplotypes and AGEs (1).

**FIGURE 3:**
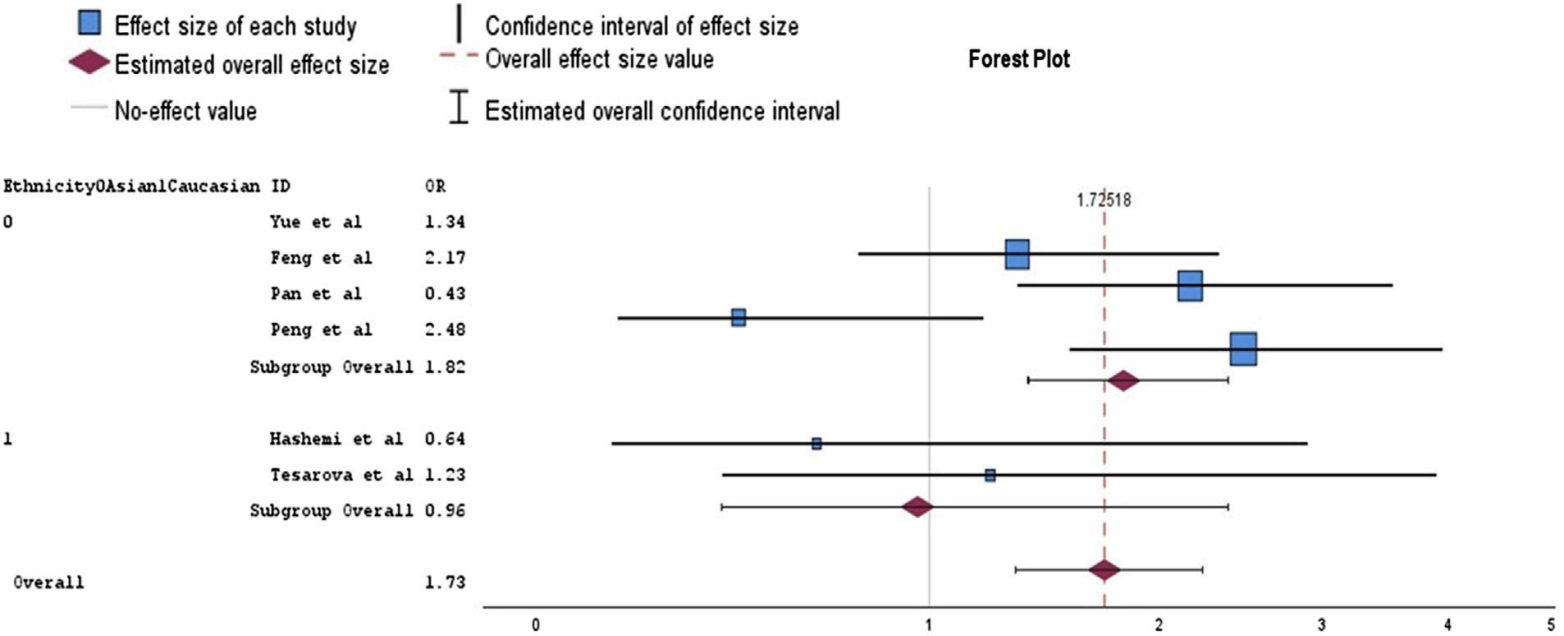
Forest plot of analysis for the association between *rs1800624* polymorphism and breast cancer in a random effects model (recessive model).

Our research included also the study by Peng et al., as it is more recent and similar to those included in Zhang et al.‘s meta-analysis. Consequently, we performed an analysis using the data sets from both studies. In our meta-analysis, none of the genetic models presented a statistically significant association with BC risk (Table 1). Subgroup analysis based on ethnicity, conducted on the recessive model—which showed less variability—indicated that BC risk was significantly increased in the Asian subgroup (OR = 1.82, 95% CI = 1.38–2.39, *p* = 0.00). However, this analysis also revealed increased heterogeneity (I^2^ = 62%), suggesting substantial variability in study results that could be attributed to different study populations, methodologies, or other factors (Figure 3). There was a borderline significant protective effect observed in the dominant Caucasian model (OR = 0.65, *p* = 0.05), with no heterogeneity (I^2^ = 0%), indicating that the studies were consistent in their findings.

**TABLE 1:** Meta-analysis results of overall genetic models.

Genetic model	Pooled ORs (95%, CI)	P-value	Heterogeneity test		Analysis model	*Z* test
			*I* ^*2*^ (%)	*P* for Q test		
Homozygous Overall	1.37 (0.82–2.31)	0.23	67	0.02	REM	1.20
Heterozygous Overall	0.99 (0.67–1.46)	0.96	80	0.00	REM	−0.05
Dominant Overall	1.00 (0.67–1.49)	0.99	82.6	0.00	REM	0.01
Dominant Asian	1.20 (0.76–1.90)	0.44	85.5	0.05	REM	0.01
Dominant Caucasian	0.65 (0.43–0.99)	0.05	0.0	0.05	REM	0.01
Recessive Overall	1.08 (0.92–2.31)	0.37	0.00	0.53	FEM	0.89
Recessive Asian	1.82 (1.38–2.39)	0.00	62	0.02	FEM	4.09
Recessive Caucasian	0.96 (0.38–2.39)	0.00	62	0.02	FEM	4.09

OR, odds ratio; CI, confidence interval; REM, random-effects model; FEM, fixed-effects model.

#### 3.1.2 Soluble RAGE

The secreted isoform of RAGE, termed soluble RAGE (sRAGE) was found to be released from cells to bind to RAGE ligands without stimulating intracellular signal transduction. As a result, the adverse effects of RAGE-signaling. Therefore, sRAGE represents a naturally occurring competitive inhibitor of RAGE-mediated events (1, 7). Receptor ectodomain shedding or splice variant [endogenous secretory (es) RAGE] secretion may generate circulating sRAGE (10, 11). Studies report decreased sRAGE levels in cancer patients, including BC, compared to controls (1, 11). Both sRAGE and esRAGE may serve as biomarkers or endogenous protection factors against RAGE-mediated pathogenesis (10, 11). As biomarkers they could potentially help assess the severity of disease or the response to therapeutic intervention. Moreover, AGEs/sRAGE levels might serve as a factor to assess the individual’s risk to develop cancer associated with early recognition (1, 7, 11).

#### 3.1.3 RAGE in breast cancer

RAGE effects are strongly dependent on the cell type and the context (physiological or pathological). Therefore, RAGE activation may not be restricted only to pathological situations, the receptor being involved in tissues homeostasis and regeneration repair upon acute injury and in resolution of inflammation (10). Some recent studies have reported a correlation between RAGE and different human pathologies including diabetes, neuronal degeneration, inflammation, Alzheimer’s disease, cardiovascular disease and cancer (2, 11). Thus, the dysregulation of the balance between pro- and anti-inflammatory signals fuels chronic inflammation, which leads to onset and development of the above-mentioned conditions. Furthermore, it is widely recognized that chronic inflammation contributes to shaping a supportive microenvironment for tumor growth and development (11, 16).

A study by Radia et al (2) on cellular cultures using breast cancer cell lines (MCF-7, SK-Br-3 and MDA-MB-231) demonstrated RAGE mRNA and protein expression, with significantly higher levels of RAGE for MD-MBA-231 (triple negative aggressive subtype). Furthermore, the knockdown of RAGE in different subtypes of breast cancer cell lines, using siRNA has a profound effect on proliferation leading to a significant increase in the number of cell in G0/G1 phase and a significant decrease of NF-kB p65 expression.

These findings concurred with Nasser et al (3), who observed that RAGE is overexpressed in a panel of aggressive breast cancer cell lines and TNBC (triple negative breast cancer) tissue. Moreover, in a study on tissue cultures, high RAGE expression, present in lymph nodes and distant metastases, was associated with poor prognosis in breast cancer (5). RAGE expression levels were higher in metastatic deposits in lymph nodes compared to primary breast cancer tumors. Furthermore, there was a significant correlation between tumor grade and RAGE intensity and scores in both primary tumors and metastatic lymph node deposits. High-grade invasive breast cancer showed greater RAGE parameters than low-grade invasive breast cancer. These findings indicate the potential of RAGE as a biomarker for tumor aggressiveness in breast cancer (9).

### 3.2 AGEs and AGE-RAGE axis in breast cancer

AGEs are a group of heterogenous macromolecules formed during non-enzymatic glycation/glycoxidation of the reactive carbonyl group of reducing sugars with free amino groups of proteins. This process is called the Maillard reaction and it is also responsible for the glycation of lipids and nucleic acids (6, 7). Although this glycation reaction is a non-specific reaction, proteins undergoing this modification suffer substantial functional and structural consequences such as: altered enzyme activity, immunogenicity, decreases ligand binding and extracellular matrix modification (4, 6, 7).

Formation of AGEs occurs endogenously in the body under physiological conditions during aging throughout life span, but also under pathological conditions associated with increases protein glycation such as cardiovascular disease, Alzheimer’s disease and diabetes (6, 7). Highly reactive α-dicarbonyl compounds [e.g., methylglyoxal (MGO) and glyoxal (GO)] are major precursors of AGEs. These compounds arise from different pathways including the Warburg effect occurring in cancer cells (7, 11, 16). MGO is considered a genotoxic agent capable of producing oxidative damage to DNA and DNA adducts as well, thus promoting tumor development and progression by accumulating in cancer cells (11, 16).

Besides being endogenously formed, AGEs occur exogenously in food, especially by thermal processing (7). Therefore, an unhealthy diet high in sugar, fat and highly processed foods along with a sedentary lifestyle also contribute to the AGE accumulation pool which leads to chronic disease development and complications (18). AGEs inversely accumulate in our tissues causing pathogenic effects on organ homeostasis, genetic fidelity, protein function and cell signaling cascades (22). The cellular effects of AGEs are mainly mediated through RAGE. RAGE expression is low in most healthy adult tissues, but also in benign breast lesions (4, 9). However, RAGE expression is induced by high glucose, reactive oxygen species (ROS), external stress, hypoxia, pro-inflammatory mediators and AGEs (6). A growing body of evidence has suggested a relationship between metabolic disorders, such as obesity, hyperglycemia or type 2 diabetes mellitus (T2DM), both increasing breast cancer risk and tumor-related mortality (8, 11).

In recent years several studies aimed to investigate the biological effects of AGEs-RAGE in promoting invasion and metastasis of breast cancer, in patients with diabetes, by using methylglyoxal-derived bovine serum albumin AGEs (MG-BSA-AGEs).

MG-BSA-derived AGEs increase cell proliferation, migration and invasion of MDA-MB-231 cell line (6, 23), whereas MCF-7 estrogen receptor (ER) positive cell proliferation and migration slightly increase without affecting cell invasion (4). Increased cell invasion is associated with enhancement of matrix metalloproteinase-9 (MMP-9) gelatinase activity, a gelatine involved in the degradation of type IV collagen of the basement membrane (6). Furthermore, MG-BSA-AGEs increase the expression of RAGE and ERK1/2 phosphorylation in different breast cancer cell lines (4, 6, 23), alongside increased expression of ER-α and ER-β without active forms of MMP-9/MMP-2 in ER positive cell lines (4). The activation of RAGE by AGEs also induces downstream signaling such as toll-like receptor 4 (TLR4)/myeloid differentiation factor 88 (MyD88), which further triggers the activation of NF-kB (23). Blocking RAGE using a neutralizing RAGE antibody reverses the MG-BSA-AGEs induced biological effects in all breast cancer cell lines (4, 6). In addition, TLR4 silencing significantly suppressed the effects of AGE-BSA on MDA-MB-231 cells (23).

Interleukin 8 (IL-8), part of the CXC chemokine family, is crucial in promoting prometastatic effects across various tumor types, including breast cancer. The conditioned medium from AGEs-exposed cancer-associated fibroblasts (CAFs) triggers paracrine activation of IL-8/CXCR1/2, leading to increased migratory and invasive characteristics in MDA-MB-231 breast cancer cells (8). Another study revealed that, besides increasing AKT and ERK phosphorylation, BSA-AGE treatment of MCF-7 cell lines determines the phosphorylation of ser118 and ser 167 within the ligand-independent activation domain of ERα. Furthermore, exposure to AGEs in tamoxifen treated MCF-7 cell lines restored proliferation in a dose-dependent manner suggesting a mechanistic link between AGEs and ERα regulation and tamoxifen efficacy (22). In contrast to previous findings, Nass et al. noted that high levels of Nε-carboxymethyl lysine (CML) in ER-positive carcinomas were associated with better relapse-free survival (RFS) outcomes when treated with tamoxifen. ER-negative cases showed poorer RFS outcomes following chemotherapy. Additionally, CML accumulation in tumors was positively correlated with the presence of ERα, postmenopausal status, and patient age, while a negative correlation was observed in grade III carcinomas and triple-negative breast cancer (5). Metastasis incidence significantly correlates with serum AGE concentrations in breast cancer patients. Diabetic patients significantly shorter metastatic interval (23).

At present, only a few prospective cohort studies evaluated the association of dietary AGE (dAGE) intake and breast cancer risk and mortality. Dietary AGEs are measured in food mainly through their metabolite CML, which can also be measured in serum by ELISA and in tissue with immunohistochemistry (24). There are several emerging dAGE databases, however the most utilized database to estimate total CML-AGE intake was developed by Uribarri et al. (25). Peterson et al demonstrated that the highest quintile of AGE intake was associated with increased risk of breast cancer in postmenopausal women after adjusting for risk factors and fat and meat intake (24). Moreover, in the Prostate, Lung, Colorectal and Ovarian Cancer screening trial (PLCO) higher CML-AGE intake was associated with increased risk of breast cancer among all women. The increased risk was more prominent in non-Hispanic white women and women diagnosed with *in situ* and hormone receptor positive breast cancer (26). Another recent study examined the association between post-diagnosis dietary CML-AGE intake and mortality among postmenopausal women. Higher CML-AGE intake after breast cancer diagnosis was positively associated with all causes, breast cancer specific and cardiovascular disease mortality. This association persisted after additional adjustment for red and processed meat intake and was particularly strong for women with hormone receptor negative breast cancer (25).

In all the above-mentioned studies, women (from Western countries) with the highest quintile of dietary AGE intake were more likely to have younger age at diagnosis, obesity or higher body mass index (BMI), be current smokers, less physically active and higher intake of total fat and meat (24–27). In Iranian women, breast cancer rates also increased with higher tertiles of dietary AGEs intake, despite their differing eating habits and dietary patterns compared to Western countries (27). Table 2 summarizes the effects of highest quintile of dietary AGEs intake.

**TABLE 2:** Characteristics of the studies evaluating dietary AGE intake.

Author	Year	Ethnicity	Sample size	Median dAGE daily intake KU/1,000 Kcal	Median follow-up (years)	Highest quintile of AGE intake and BC risk/mortality	Nr of BC cases	P-value
(24)	2020	Non-hispanic white 90%	183,548	Prediagnosis – 5,932 (range 845,2–3836,5)	12,8	Risk - HR 1,09 95% CI −1,02–1,16	9,851	*p* = 0,03
(24)	2020	Non-hispanic white 90%	183,548	Prediagnosis – 5,932 (range 845,2–3836,5)	12,8	Advanced stage risk – HR 1,37 95% CI −1,09–1,74	9,851	P < 0,02
(26)	2020	Non-hispanic white 90%	27,464	Prediagnosis – 6,105 ± 2,691 (range 867–43,387)	11,5	Risk - HR_Q5 vs Q1_ 1,30 95% CI −1,04–1,62	1,592	P-trend 0,04
(25)	2021	Non-hispanic white 90%	161,808	Postdiagnosis – 6,659 ± 2,309 (range 830–19,420)	15,1	BC mortality -HR_T3 vs T1_ 1,49, 95% CI −0,98–2,24	2023	P-trend 0,29
(27)	2023	Iranian 100%	401	9,251 (range 7,450–11,818)	5	Risk OR 2.29 95%CI −1.19–4.39	134	P-trend 0,012

Due to the diversity of AGEs, existing dietary AGEs databases may not accurately reflect total exposure levels or the overall burden of exogenous AGEs in the body. A large case-control study among the Chinese population found that higher concentrations of AGEs and the AGEs/sRAGE ratio were linked to an increased risk of breast cancer, regardless of molecular subtype. In contrast, sRAGE levels were inversely associated with breast cancer risk, particularly in individuals under 60 years old. Additionally, the highest quartile of AGEs was associated with a greater proportion of deaths, indicating the potential prognostic value of AGEs levels (1).

Another study suggested that pigment epithelium-derived factor (PEDF). May exert antitumor effects in AGE-exposed MCF-7 breast cancer cells. This adipocytokine with multifaceted functions could suppress NADPH oxidase-induced ROS generation, RAGE gene expression, VEGF (vascular endothelial growth factor) expression and MMP-9 expression via interaction with laminin receptor, Moreover, decreased levels of PEDF expression in breast cancer tissue were linked to increased growth, aggressiveness, and metastasis (28).

### 3.3 HMGB1 and HMGB1- RAGE axis in breast cancer

High mobility group box 1 (HMGB1) is an extremely versatile protein, located predominantly in the nucleus of quiescent eukaryotic cells (29). HMGB1 is a 215 amino acid long protein that consists of two DNA-binding domains (HMG A box and HMG B box) and a C-terminal acidic tail (30), which encompasses RAGE and TLR binding sites (29). Expressed in all vertebrate nuclei (31), HMGB1 is released from cells either passively during cell death (necrosis) or actively by activated immune cells or upon cytokine stimulation (14, 15, 29). The subcellular localization of HMGB1 influences its regulatory role in normal physiological and pathological processes. Acetylation appears to be the key modification that impacts HMGB1 localization (15, 31). Usually localized in the nucleus, HMGB1 acts as a DNA binding protein, maintaining DNA structure and genome stability, or interacting with transcription factors to exert co-activator or co-repressor activity through its DNA-binding domains (29, 30, 32, 33). As an abundant non-histone component of chromatin (31), HMGB1 can recognize and bind with high affinity to distorted DNA and induce kinks in linear DNA fragments (14).

In the cytoplasm, HMGB1 is mainly associated with the regulation of autophagy in cancer cells (30), a degradation of dysfunctional organelles and proteins to generate metabolic fuels during starvation (14). Activated monocytes prevent HMGB1 from relocating to the nucleus. Therefore, HMGB1 is involved in modulating cell stress response. It also inhibits apoptosis while promoting autophagy by binding to beclin-1 and disrupting the autophagy-inhibitory interaction between BECH1 and BCL2 (29, 32, 34). Cytoplasmatic HMGB1 can either leave the cell through loss of membrane or via active secretion (29).

HMGB1 can be released from necrotic cells, but not from apoptotic cells (14), as apoptosis is a programmed mechanism of cell death that is cleared by phagocytosis and escapes from inflammatory surveillance (35). Activated macrophages secrete HMGB1 actively, either in a partially tumor necrosis factor (TNF)-dependent manner or in response to various inflammatory and angiogenic signals (14). Therefore, tumor-associated immune cells, as well as cancer cells themselves, can release high levels of the alarmin within the tumor microenvironment (16).

Once released into the extracellular milieu, HMGB1 functions as a DAMP (29) and stimulates the innate immune system to recruit monocytes to inflammatory sites (30) via interactions with several pattern-recognition receptors (PRRs), namely, TLR2, TLR4 and RAGE (14, 15, 29). HMGB1 induces the release of cytokines and chemokines from immune cells. Depending mainly on ligand-receptor interaction, HMGB1 acts as a biomarker of immunogenic cell death (ICD), maturing antigen-presenting dendritic cells and enhancing their antigen-presenting capacity (30, 36). The effects of HMGB1 are dependent at least in part upon Myd88 (36, 37). However its main signaling pathway is through the interaction with RAGE (14). HMGB1-RAGE interaction results in the secretion of pro-inflammatory cytokines (TNF-α and IL-8) and enhanced expression of leukocyte adhesion molecules through NF-kB activation in endothelial cells. Consequently, inflammation is further promoted in a positive feedback loop, contributing to sustained inflammation and angiogenesis (14, 16).

The genetic predisposition of polymorphism in HMGB1 genes to breast cancer prognosis was evaluated by four studies. Yue et al evaluated three polymorphisms (rs2249825, rs1412125, rs1045411) in the HMGB1 gene in the Han Chinese population, however haplotype analysis failed to reveal any significance in breast cancer risk (20). The results of Lee at al’s study in a population of Korean women, showed that the single nucleotide polymorphism (SNP) rs143842 of the MMP2 gene was constantly associated with breast cancer prognosis in patients with disease free survival (DFS) or overall survival (OS) events. Furthermore, rs243842 and rs243867 of the MMP2 gene showed statistically significant associations with a poor breast cancer prognosis regarding DFS, while among OS patients rs243842 in the MMP2 gene and rs4145277 in the HMGB1 gene were significantly associated with poor prognosis. In contrast, rs7656411 in TLR2 and rs7045953 in TLR4 were significantly associated with a good prognosis in the OS patients (38). Moreover, another study in the Han Chinese population reported an association between patients with G allele in the rs1360485 or rs2249825 domains and the likelihood to progress to T2 tumor and lymph node metastasis. In addition, the study revealed the presence of G allele in SNPs rs1360485 or rs2249825 was associated with T2 tumor and distant metastasis amongst HER2-enriched and TNBC, whereas having 1 C allele in the rs1412125 domain increased the risk of pathologic grade 3 disease (39). Evidence from a meta-analysis revealed that rs1045411 polymorphism was positively associated with risk of breast cancer amongst Hans rather than Caucasians, with no obvious difference amongst other cancer types (40).

The dual anti-tumor and pro-tumor biological functions of HMGB1 make its precise role in breast cancer progression quite elusive. A new paradigm in cancer biology highlighted that conventional chemotherapeutic agents not only kill cancer cells by apoptosis, but also induce other forms of non-apoptotic death such as necrosis and autophagy (32, 35). Therefore, HMGB1 released from necrotic cells during chemotherapy may stimulate (through RAGE) the proliferation of the remnant cancer cells and metastasis contributing to the resistance to cancer therapy (14, 32). The production of high levels of HMGB1 by tumor cells, as well as by infiltrating inflammatory cells, favors the establishment of a highly immunosuppressive tumor microenvironment. Thus tumor cell proliferation and progression are enhenced (29). However, a more recent study found significantly reduced serum levels of HMGB1and E-cadherin after neoadjuvant chemotherapy (41).

Thus, targeting RAGE and HMGB1-mediated signaling pathways could be an innovative approach for therapeutic intervention and prevention strategies in breast cancer management. Dhumale et al. found that quercetin, a natural flavonoid, effectively inhibits MCF-7 cell proliferation by downregulating RAGE and HMGB1 mRNA and protein levels while inhibiting NF-kB activation. Additionally, quercetin demonstrated a protective role against necrotic injury and enhanced apoptotic cell death in the MCF-7 cell line (35). HMGB1 silencing promotes apoptosis, without affecting proliferation (42).

Despite advancements in early detection and treatment of breast cancer, distant relapse remains a challenge, prompting studies to identify prognostic markers for metastasis risk. Ladoire et al. explored the association between nuclear HMGB1 and cytoplasmic light chain 3B (LC3B) puncta in two cohorts of breast cancer patients. They found that patients with favorable prognostic factors (tumor size <2 cm, N+ <3, positive HR status, tumor grade < III) had a low risk of metastatic relapse, regardless of LC3B and HMGB1 expression. Although double positivity for LC3B and HMGB1 was linked to better metastatic-free survival (MFS), it served as a useful stratification tool in high-risk patients (34). Additionally, another study demonstrated a strong correlation between cytoplasmic LC3B and nuclear HMGB1 expression with local immune parameters. LC3B correlated only with CD8^+^ cytotoxic T lymphocytes whereas, HMGB1 correlated with both local and peritumoral infiltrates involving FOXP3+ regulatory T cells and CD68^+^ tumor-associated macrophages (43). CAFs are the most abundant cell type in the breast cancer microenvironment and their abundance correlates with cancer progression, metastasis and poor prognosis (30, 44). They secrete extracellular matrix to induce cancer cell growth and metastasis and act as an important paracrine source that regulates cancer hallmarks (45). Autophagic CAFs release HMGB1, which activates TLR4 to enhance tumorigenicity (46). HMGB1 cand also be liberated in the microenvironment by either active secretion from stressed cells or passive release from damaged or dying cells (44), functioning as a multifunctional cytokine (45).

Recent studies have highlighted a bidirectional interaction between breast cancer cells and CAFs. Amornsupak et al. examined the clinical implications of α-smooth muscle actin positive (ASMA+), a marker of activated fibroblasts, and the expression of HMGB1 and RAGE in breast cancer. Their results showed that high ASMA + expression was significantly correlated with larger tumor size, clinical stage III-IV, and angiolymphatic and perinodal invasion. Also increased cytoplasmic HMGB1 was significantly associated with smaller tumor size, earlier stages, luminal subtype, and hormone receptor expression. These findings suggested that both markers are independent predictive factors. Furthermore, the risk of metastatic relapse was notably higher in patients with ASMA + high/HMGB1 low in non-inflammatory breast cancer samples (44). Chen et al. reported that HMGB1 secreted by breast cancer cells activates fibroblasts through RAGE, with higher expression and secretion levels of HMGB1 seen particularly in highly migratory and invasive breast cancer cell lines. The activated fibroblasts then enhance breast cancer cell metastasis by increasing lactate production, creating a microenvironment with a lower pH that accelerates cancer cell motility (47). In addition, the interaction between HMGB1-RAGE promotes breast cancer cell invasion through PI3K/AKT signaling pathway. This fundamental signal transduction network contributes to cell survival, cell growth and cell progression (32). In a PI3K/AKT dependent manner, programmed death ligand 1 (PD-L1) expression is also increased, which leads to the destruction of the effector T cells (45). The knockdown of HMGB1-RAGE-PI3K/AKT pathway could attenuate breast cancer cell aggressive phenotypes (45) and overcome resistance to anti-tumor treatment (32). Activation of the RAGE/multiligand axis can strongly influence cell invasion through enhancement of epithelial-to-mesenchymal transition (EMT). This form of trans-differentiation of epithelial cells into mesenchymal phenotypes allows solid tumors to become more aggressive (16). High metastatic TNBC tumors possess the traits of harboring epithelial-mesenchymal plasticity, which could initiate tumor cells migration. Such tumors also express lower level of miR (microRNA)-205 inversely associated with tumor stage and distal metastasis of TNBC. Overexpression of miR-205 could suppress the cell growth and EMT biological features of TNBC cell partially through direct targeting HMGB1/RAGE (48). Table 3 summarizes HMGB1 functions in BC.

**TABLE 3:** HMGB1 functions in BC.

Mechanism of action	Effects	References
HMGB1 acts as a DNA binding protein	Maintains DNA structure and genome stability, interacts with transcription factors	(29–33)
HMGB1 regulates autophagy	Prevents apoptosis and promotes autophagy by binding to beclin-1	(29, 32, 34)
Activated macrophages secrete HMGB1	Involved in modulating cell stress response and inflammation	(14, 15, 29)
HMGB1 as a DAMP	Stimulates innate immune response, recruits monocytes to inflammatory sites	(29, 30)
HMGB1 enhances tumor-associated immune response	Matures antigen-presenting dendritic cells, enhances their antigen-presenting capacity	(30, 36)
High levels of HMGB1 promote tumor proliferation	Supports establishment of a highly immunosuppressive microenvironment	(29)
Interaction between HMGB1 and TLR/RAGE	Induces cytokine release, activates NF-kB, promotes inflammation and angiogenesis	(14, 16, 30)
HMGB1-RAGE-PI3K/AKT pathway activation	Enhances breast cancer cell invasion and PD-L1 expression, leading to immune evasion	(45)

### 3.4 S100 protein family and S100 proteins - RAGE axis in breast cancer

In humans, the S100 protein family consists, currently, of 25 known members (49) of calcium-binding low-molecular weight proteins which possess two Ca^2+^ binding domains: a carboxyterminal canonical EF-hand and an amino-terminal pseudo-EF-hand, connected by a “hinge” region (50). This family of proteins modulates cellular responses by functioning both as intracellular Ca^2+^ sensors and as cytokine-like extracellular factors interacting with receptors (49, 50). Within the cells they participate in the regulation of proliferation, differentiation, migration, invasion, apoptosis, calcium homeostasis and inflammation (14, 50). Moreover, the S100 protein family is also crucial to aerobic glycolysis and lymphocyte recruitment (51). Some family members demonstrated a role in innate immunity (52). Several S100 proteins may be released or secreted extracellularly and regulate cell functions in an autocrine and paracrine manner via different cell surface receptors, including RAGE and TLR4 (14). They may play a role in different stages of tumorigenesis as cancers exhibit a distinctive S100 protein profile that can be both stage specific and subtype specific (50). The promotion of proliferation by S100 proteins is also often mediated in a RAGE dependent manner, inducing NF-kB and MAPK signaling (an important bridge in the switch from extracellular signals to intracellular response (49).

A growing body of evidence has reported the overexpression of several S100 family members in breast cancer cells, whereas normal breast tissues lack S100 protein overexpression (50, 53, 54). Furthermore, S100 gene expression correlates to clinical-pathological features (molecular subtype, ER status, grading) and survival data (OS, DFS). Significantly higher expression levels are observed in ER negative, higher-grade tumors and basal like or HER2 expressing tumors, whereas luminal A and liminal B tumors harbour lower expression levels (52).

S100P expression is significantly higher in BC tissue than in benign fibroadenoma, promoting the aggressive properties of breast cancer cells and metastasis (53, 55). The binding of extracellular S100P to RAGE upregulates NF-kB activity (50). This may represent a compensatory mechanism of cell survival and proliferation in ER positive cell lines resistant to tamoxifen, as S100P expression level is elevated (55). Moreover, S100P is also involved in resistance to targeted therapies through the RAS/MEK/MAPK pathway, therefore inhibition of S100P could lead to reversing the trastuzumab resistance (55).

S100A7 (psoriasin), a member of the epidermal differentiation complex, is widely overexpressed in invasive ER negative breast cancer (56) especially in lymph node metastasis (57). Expression of S100A7 in breast tumors negatively impacts prognosis through enhancing proliferation, production of proinflammatory molecules (IL-1α, CXC-L1, CXC-L8) (56) and tumor-associated macrophages (TAMs) recruitment (49, 56). Furthermore, by binding to RAGE, the proinflammatory ligand S100A7 induces breast cancer growth and metastasis leading to ERK, NF-kB activation and cell migration (49, 58). RAGE/S100A7 interactions conditions the tumor microenvironment by increasing the recruitment of MMP9-positive TAMs (58). S100A7 is also secreted by tumor cells (50) increasing ROS and VEGF expression through RAGE during tumorigenesis and enhancing tumor progression by promoting oxidative stress responses and angiogenesis (16, 56). Additionally, lipopolysaccharide (LPS), derived from the breast tumor microbiota, was found to increase S100A7 expression in breast cancer cells *in vitro*. This suggests that the commensal microbiota in breast tissues may contribute to tumor growth through a novel LPS/S100A7/TLR4/RAGE axis. Consequently, elevated levels of S100A7 and reduced expression of TLR4 could serve as indicators of poor prognosis for invasive breast cancer (59).

Patients with metabolic disorders (obesity, T2DM) demonstrate increased breast cancer mortality in association with an altered expression profile of both RAGE and the IGF (insulin growth factor)-1/IGF-1R (receptor) axis (16, 60). In ER positive breast cancer cells, these alterations favor STAT3-dependant transcriptional activation of the S100A7 gene. S100A7/RAGE dependent activation of human vascular endothelial cells towards the acquisition of pro-angiogenetic phenotype is enhanced (60). In ER positive breast cancers S100A7 correlated with worse prognosis parameters and higher tumor grade (60). On the other hand, previous studies reported that in ER positive breast cancer cells, S100A7 inhibits proliferative capacity by degradation of β-catenin (50) and inhibits migration and invasion by inactivating MMP9-secretion (57).

S100A4 overexpression in breast cancer cells provides increased migratory capacity (50) as the interaction with MMP2 induces EMT (49). Stromal cell-derived S100A4 modulates the tumor immune response (50), consequently to high expression in stromal cells of the tumor microenvironment (fibroblasts, T-cells, macrophages) (49). Therefore, S100A4 contributes to the functional liaison between cancer cells and the surrounding microenvironment to ward worse outcome (61). Extracellularly, in the tumor microenvironment, S100A4 interacts with RAGE in a paracrine manner (61, 62), This interaction subsequently induces the release of pro-inflammatory factors IL-6, IL-8 and CXC-L10 which then convert monocytes into TAMs (49). A pro-tumoral response is elicited in the tumor microenvironment (50, 63), including geminin–overexpressing TNBC cell (64). S100A4 monoclonal antibodies significantly limit breast tumor invasion and metastasis *in vivo* (39, 65) and bone loss caused by breast cancer bone metastasis (66). S100A4 knockdown diminishes stanniocalcin 1-induced lung metastasis of breast cancer (67).

In addition, TNBC cells show higher expression levels of fibroblast growth factor 2 (FGF2) and S100A4 than non-TNBC patients. Upon FGF2 treatment, the paracrine activation of the S100A4/RAGE pathway triggers angiogenic effects in vascular endothelial cells and promotes the migration of CAFs (61). Recent evidence indicates that apoptotic cell death can be utilized by nearby tumor cells to promote metastatic capabilities. In this context, chromatin-bound S100A4 is expelled from apoptotic metastatic breast cancer cells, which then activates RAGE receptors in neighboring surviving tumor cells. This activation leads to ERK signaling and supports metastatic growth (68).

S100A9 is primarily expressed in immune cells (myeloid cells, neutrophiles) (49). S100A8 and S100A9 naturally form a stable heterodimer (51, 69), participating in myeloid cell differentiation (69, 70). This heterodimer is crucial for the formation of pre-metastatic niche at different organ sites (69). Stromal S100A9 localization correlates to parameters such as large tumor size, HER2 positivity and nodal stage in ER-negative/PR-negative breast cancers (71). S100A8/S100A9 high mRNA expression was correlated with lower OS in all breast cancer types (69), especially in Her2 positive and TNBC subtypes (70).

Extracellularly, S100A8/S100A9 heterodimer fulfils the characteristics of a DAMP (49) and binding with RAGE, through MAPK pathway, contributes to the viability and migration of tumor cells in a concentration-dependent manner (51). Moreover, S100A8/S100A9-RAGE interaction stimulates NF-kB signaling, which results upregulation of cytokines and pro-inflammatory, such as IL-1β and tumor necrosis factor α (TNF-α) (49).

Overexpression of S100A9 inhibited antitumoral immunological activity in the tumor microenvironment via promoting tumor cell metabolism in HER2 positive breast tumors (51). Furthermore, miR-185-5p inhibited the S100A8/A9 induced EMT of breast cancer cells by the NF-kB/Snail signaling pathway and was negatively associated with RAGE (72). In addition to modulating the tumor microenvironment and cell metabolism, S100A8/S100A9 enhances chemoresistance of breast cancer cells by activating pro-survival ERK1, ERK2 and ribosomal protein S6 kinas β1 pathways (69). BC cells may induce substantial molecular changes in non-tumorigenic mammary epithelial cells via dynamic cell–cell interactions through S100A8/S100A9 (73). BRCA1 deficiency activates S100A9-CXCL12 signaling for cancer progression and triggers the expansion and accumulation of myeloid-derived suppressor cells, creating a tumor-permissive microenvironment and rendering cancers insensitive to immune checkpoint inhibitors (74). Non-invasive detection and measurement of exosomal S100A8/A9 release in potential pre-metastatic sites would strongly promote the clinical utility of this marker (75).

A few recent studies have reported that women with breast cancer who experience greater distress (76) or have lower social support and social wellbeing (SWB) (77) displayed greater inflammatory signaling and poorer clinical outcomes as well as greater S100A9/S100A9 levels (77). In this context, some studies suggested that reducing distress secondary to stress management techniques or improving SWB could modulate inflammation which may influence disease outcomes through RAGE-mediated processes and decrease S100A8/S100A9 levels (76, 77). S100A8 and its cognate-binding partner S100A9 exhibit potential prognostic biomarkers for reactivation of dormant tumor cells, prediction of metastatic risk and therapeutic responses failure in several malignancies, including BC (70).

Furthermore, S100A14 associates with the clinical outcome of breast cancer patients (54) as it significantly correlates with lymph node metastasis (78) and reduced OS (69, 78) especially in the luminal B subtype (69). To promote migration and invasion of breast cancer cells, S100A14 requires functional p53 to affect MMP2 transcription (54, 79) while promoting metastasis via RAGE-NF-kB pathway (54). Moreover, S100A14 plays an important role in HER2 induced proliferation, functioning as a modulator of HER2 signaling (54). Altogether, S100A14 appears to be a potential biomarker for a better selection of high-risk patients, especially in the Her2 positive setting. The complex mechanisms of action regarding S100 proteins in breast cancer are summarised in Table 4

**TABLE 4:** S100 protein mechanisms in BC.

S100 protein	Mechanism of action	Tumor type/Correlation	References
S100P	Upregulates NF-kB activity via RAGE, involved in resistance to targeted therapies, contributes to trastuzumab resistance	Worse prognosis in invasive ductal carcinoma, tamoxifen resistance	(50, 53, 55)
S100A7	Enhances proliferation and proinflammatory molecules through RAGE, recruits TAMs, increases ROS and VEGF	Associated with ER-negative breast cancer and lymph node metastasis	(49, 56, 58)
S100A4	Induces epithelial-mesenchymal transition (EMT) via MMP2, promotes pro-tumoral response, stimulates angiogenesis through RAGE	Associated with increased migration, TNBC and worse outcomes	(49, 50, 61)
S100A8/S100A9	Promotes formation of pre-metastatic niches, stimulates NF-kB signaling via RAGE, enhances chemoresistance	Correlates with HER2-positive and TNBC subtypes, lower OS	(49, 51, 69, 70)
S100A14	Promotes migration and invasion via RAGE-NF-kB pathway, modulates HER2 signaling	Correlates with lymph node metastasis, reduced OS in luminal B subtype	(54, 69, 78)

The implications of the association between RAGE and its ligands and breast cancer progression, as well as their potential role as biomarkers for identifying high-risk patients, were further examined in the discussion section. We particularly addressed the potential for new drug development and metastatic risk of breast cancer patients.

## 4 Discussions

Our literature research reveals that breast cancer remains a highly diverse entity. Its morphological, molecular, and biochemical features impact disease progression, prognosis, and therapy response (4). Metastasis, especially in TNBC, leads to high mortality. Targeting mechanisms that regulate metastasis is crucial (9). RAGE is a multiligand receptor involved in cancer, immune, and metabolic diseases. It contributes to metastasis by promoting cell transformation and a supportive tumor microenvironment through ligands such as AGEs, HMGB1, and S100 proteins (7, 10, 16). Therefore, targeting RAGE and its ligands could be novel strategies for clinical intervention in the treatment of breast cancer patients.

Polymorphisms like rs1800624 show inconsistent associations with breast cancer risk, with some studies showing decreased risk (18, 19), and others, especially among Asians, reporting increased risk (13, 17, 20). The Gly82Ser variant (rs2070600) may raise cancer risk by lowering circulating sRAGE (80). As a result of the available studies using various genotyping methods, different sample sizes and different ethnicities, the accuracy and reliability of the conclusions may be biased. Thus, the role of individual genetic variations remains unclear. To strengthen the findings and deepen the investigations of various ethnic groups, further large-sample studies are warranted.

Diabetes and hyperglycemia accelerate AGEs accumulation, which increases breast cancer proliferation, migration, and invasion (6, 7). AGEs activate RAGE-related pathways (ERK, MAPK, STAT3) and enhance MMP-9 activity, especially in ER-negative MDA-MB-231 cells (4, 23). In contrast, ER-positive MCF-7 cells show no MMP activation, underscoring their lower invasive potential (4). AGE-BSA exposure increases phosphorylation of RAGE, p70S6K1, STAT3, p38 MAPK, TLR4, and MyD88 in MDA-MB-231 cells (4, 23). Additionally, the RAGE-lysophosphatidic acid (LPA) axis promotes EMT markers (vimentin, Slug, Twist) and RAGE-dependent EphrinB2/EphA3 transduction signaling enhances migration in BC cells (81, 82). Inhibition of RAGE using neutralizing antibodies or silencing TLR4 has shown therapeutic potential (4, 6, 23). Blocking RAGE in CAFs may reduce tumor growth driven by insulin signals, as evidenced by the cross-talk between RAGE and insulin receptors (83). RAGE antagonists like RP7, TTP488, and FPS-ZM1 show promise in reducing TNBC metastasis by inhibiting NF-kB and ERK pathways, with TTP488 showing a favorable safety profile (84, 85). RP7 suppresses ERK1/2, blocks NF-kB, and downregulates HMGB1 to induce apoptosis (84). Although the previously mentioned RAGE inhibitors have shown promising results in reducing cell growth and invasive potential of breast cancer cells *in vitro* and *in vivo* on murine models, further research is required. As TTP488 displays a favorable safety profile in human studies, this provides rationale for evaluating RAGE inhibitors in clinical trials to treat metastatic breast cancer or to prevent metastatic spread in high-risk breast cancer patients. Further clinical trials are needed for these RAGE inhibitors as chemotherapy remains the primary option for TNBC patients (85). Additionally, phosphorylation of p70S6K1 and residues ser118 and ser167 after AGE exposure may serve as biomarkers for tamoxifen resistance (6, 22). IL-8 expression is upregulated in T2DM patients with breast cancer and blocking IL-8 could reverse treatment resistance and halt tumor progression (8). Early detection of therapy resistance, by evaluating the above-mentioned biomarkers could prompt tailoring treatment strategies to overcome resistance and improve patient outcomes, regardless of the adjuvant or metastatic setting.

AGEs, which are common in Western diets high in sugar, protein, and fat, may increase breast cancer risk. Detecting plasma AGEs is a better marker for evaluating total exposure (1, 24–26). Although dietary AGEs, particularly CML, are difficult to assess accurately (1), AGEs may influence early mammary morphogenesis during puberty, leading to an increased breast cancer risk later in life (86). Monitoring dietary AGEs and serum levels (AGEs or AGEs/sRAGE ratio) may offer new prevention strategies alongside current screening procedures, especially for high-risk populations (obese, diabetic, sedentary women). For example, serum AGEs levels could be used to intensify in patients <60 years old (1). As diet and lifestyle are modifiable breast cancer risk factors, it could be equally important to implement early intervention strategies to reduce dAGEs intake during screening for breast cancer and during follow-up after breast cancer diagnosis.

Concerning HMGB1further research is warranted to better understand the exact mechanism through which it influences metastasis because of its dual role in breast cancer. HMGB1 acts as a tumor promoter outside the cell and modulates inflammation through the RAGE and TLR pathways, whereas intracellularly binding to DNA to maintain nuclear integrity (14, 35, 45). It promotes cancer progression by activating PI3K/AKT and NF-kB pathways, contributing to metastasis and therapy resistance (30). HMGB1 also induces breast cancer-associated bone pain via RAGE binding to sensory neurons (87). Several studies suggested the potential for therapeutic intervention in targeting RAGE and HMGB1-mediated signaling pathways as a way of overcoming resistance to cancer therapy, primarily chemotherapy (32, 35). Conversely, a more recent study proposes plasma HMGB1 followed by E-cadherin as potential biomarkers to predict therapeutic response to neoadjuvant chemotherapy (41). Moreover, HMGB1 gene polymorphisms may influence tumor size and metastasis, particularly in Han Chinese populations (39, 40). Cytoplasmic HMGB1, alongside markers like ASMA^+^ and LC3B, could be used to predict metastasis risk, enhancing patient stratification after adjuvant treatment (34, 44).

S100 proteins, particularly S100A4, S100A7, and the S100A8/S100A9 heterodimer, are involved in cancer progression, inflammation, and metastasis through their interaction with RAGE (49, 56, 69). S100A4 functions in the extracellular milieu as a liaison between cancer cells and the surrounding microenvironment (61, 88) to mediate proinflammatory response (49). S100A7 and S100A9 also contribute to oxidative stress, recruitment of tumor-associated macrophages (TAMs), and pro-angiogenic signaling (58, 60). S100A7 has dual roles in ER-positive cancers, either promoting or inhibiting tumorigenesis depending on the context (57). S100A14 and S100P serve as poor prognostic markers, particularly in luminal B subtype and ER-positive tumors, respectively (52, 69). High S100A7 and cPLA2 co-expression is associated with poor survival in TNBC, suggesting potential for cPLA2 inhibitors as novel therapy (89). Chronic stress and low social support in breast cancer patients are linked to increased inflammatory signaling and higher S100A8/S100A9 levels, connecting psychological factors with inflammation and metastasis (76, 77, 90). Altogether, the S100 protein family provides various targets for potential intervention. S100A7, S100A14 and S100P could serve as prognostic markers for identifying high-risk patients and altering current therapeutic strategies for better disease control. S100A4 monoclonal antibodies could provide additional research directions leading to new drug developments, as they demonstrated efficacy in limiting tumor invasion and metastasis in murine models and cell cultures (39, 65–67). In addition, monitoring S100A8/S100A9 levels could lead to earlier intervention in managing psychological distress in metastatic patients, which was linked to increased inflammatory signaling (76, 77, 90).

Personalized approaches targeting RAGE and its ligands, along with managing dietary AGEs and psychological stress, may improve prevention and treatment strategies for breast cancer patients, particularly those with TNBC or diabetes.

## 5 Conclusion

Breast cancer represents a public health issue as many patients succumb to the disease due to metastatic spread. RAGE and its ligands emerge as new actors in breast cancer biology, however the activation of complex downstream signaling pathways and their implications in breast cancer tumorigenesis are yet to be fully explored and understood. Further studies are warranted to deepen our understanding of the mechanisms through which RAGE and its ligands enhance the metastatic potential of breast cancer cells. The available research highlights the potential of AGEs, HMGB1 and members of the S100 protein family as prognostic markers that may predict treatment outcome and metastatic risk. These markers could guide treatment selection and monitoring strategies for patients with more aggressive subtypes. Consequently, the biomarkers could aid in the development of more effective screening strategies in high-risk populations. Furthermore, silencing RAGE in human breast cancer cell lines and murine models, either by neutralizing antibody, sRAGE or miRNAs, impairs cell proliferation, migration and metastasis paving the way for further research, potentially transitioning to human studies. Clinical trials to develop potential new targeted therapies are in dire need especially for the treatment of the more aggressive subtypes of breast cancers.
